# Sexual risk behavior and pregnancy in detained adolescent females: a study in Dutch detention centers

**DOI:** 10.1186/1753-2000-1-4

**Published:** 2007-06-26

**Authors:** Sannie MJJ Hamerlynck, Peggy T Cohen-Kettenis, Robert Vermeiren, Lucres MC Jansen, Pieter D Bezemer, Theo AH Doreleijers

**Affiliations:** 1 VU University Medical Center, Dept. of Child & Adolescent Psychiatry, Amsterdam, the Netherlands; 2 VU University Medical Center, Dept. of Clinical Psychology, Amsterdam, the Netherlands; 3 VU University Medical Center, Dept. of Clinical Epidemiology and Biostatistics, Amsterdam, the Netherlands; 4 Leiden University Medical Center/Curium Academic Center for Child and Adolescent Psychiatry, the Netherlands

## Abstract

**Background:**

The purpose of this study was to investigate the lifetime prevalence of teenage pregnancy in the histories of detained adolescent females and to examine the relationship between teenage pregnancy on the one hand and mental health and sexuality related characteristics on the other.

**Methods:**

Of 256 admitted detained adolescent females aged 12–18 years, a representative sample (N = 212, 83%) was examined in the first month of detention. Instruments included a semi-structured interview, standardized questionnaires and file information on pregnancy, sexuality related characteristics (sexual risk behavior, multiple sex partners, sexual trauma, lack of assertiveness in sexual issues and early maturity) and mental health characteristics (conduct disorder, alcohol and drug use disorder and suicidality).

**Results:**

Approximately 20% of the participants reported having been pregnant (before detention), although none had actually given birth. Sexuality related characteristics were more prevalent in the pregnancy group, while this was not so for the mental health characteristics. Age at assessment, early maturity, sexual risk behavior, and suicidality turned out to be the best predictors for pregnancy.

**Conclusion:**

The lifetime prevalence of pregnancy in detained adolescent females is high and is associated with both sexuality related risk factors and mental health related risk factors. Therefore, prevention and intervention programs targeting sexual risk behavior and mental health are warranted during detention.

## Background

Sexual risk behavior and teenage pregnancy are significant problems in detained girls [1,2]. Therefore, issues related to sexuality may be an important focus for intervention and treatment during detention, as these girls may continue their sexual risk behavior after release.

High rates of sexual risk behavior and unplanned pregnancies have been noted among North American adolescent female detainees. A prevalence study among 197 adolescent female detainees found that 34% had not used any contraception in the past 2 months, 20% had had sexually transmitted diseases (STDs), and 32% had been pregnant [3].

Moreover, US studies among teenage adolescent females in the general population have demonstrated correlations between risk factors such as conduct disorders, alcohol and drug abuse and adverse psychosexual outcome such as promiscuity and teenage pregnancy [4-11]. Because these risk factors are highly prevalent in a detained population, it is no surprise that high pregnancy rates are found in this troubled population. In addition, previous research has consistently shown that early sexual trauma determines later sexual risk behavior as well as adolescent pregnancy [12-18]. Early physical maturity has been reported to be a potential risk factor for a variety of problem behaviors [19-21], as well as for teenage pregnancy, as early maturers may become sexually active at a younger age than adolescent females who mature later [22,23].

Finally, there is a relationship between suicidality and teenage pregnancy [24,25]. For those reasons, investigating correlates for teenage pregnancy in a detained population may be warranted.

Because risk factors of 'early' pregnancy in detained adolescent girls are still relatively unexplored, the main aim of the current study was to investigate the relationship with a range of potentially associated factors known from previous research in detained girls as well as in general population samples. Factors to be included are: sexual risk behavior, multiple sex partners, sexual trauma, early maturity, conduct disorder, alcohol use disorder, drug use disorder, and suicidality, as well as lack of assertiveness in sexual issues.

The first objective of this study was to investigate the lifetime prevalence of teenage pregnancy in detained adolescent females in the Netherlands.

The second objective was to explore differences between the pregnancy and the non-pregnancy group with respect to a number of variables of interest such as sexuality related characteristics, early maturity, and mental health characteristics. We expected to find differences with the above mentioned risk factors being more prevalent in the pregnancy group.

Finally, it was our objective to investigate which factors predicted pregnancy best.

## Methods

### Participants

At the time of this study, seven Juvenile Justice Institutions (JJIs; detention centers) provided closed placement for adolescent females, of which three participated in this study (covering 57% of all places nationwide). As this study covered the majority of the available places, and because females are placed in a JJI on a random basis (when a place is available), this study sample was considered representative for the population of detained girls in the Netherlands. Between September 2002 and April 2004, all newly admitted girls (N = 256) were approached for participation in their first month of detention, of whom 229 (89.5%) agreed to participate. Of the 27 non-participants, 19 (7.4%) refused participation, while another 8 (3.1%) were not able to participate because of an insufficient command of the Dutch language. Another 17 girls were excluded because they were released before or during the study, or because they had not completed the questions on pregnancy, bringing the final group included in the analyses to 212. Approximately equal numbers of participants were recruited from each of the three institutions. The age of the participants varied from 12 to 18 years (mean 15.6; SD 1.4), and ethnicity could be broken down as follows: 57.2% Dutch ethnicity, 14.6% Surinamese, 7.8% Moroccan, 3.9% Antillean, 1.5% Turkish, and 15.1% other. In 81.1% of cases, the girls had been placed in the institution under a civil law measure. Considering previous placements, 35.2% of the participants had previously been placed in a JJI, and more than 72.2% had previously undergone a residential placement of some kind (other than JJI). Considering previous care, 16.8% of the girls had a history of foster care and 74.2% had received some kind of outpatient care. In terms of the socio-economic backgrounds, about half of the mothers (48.6%) had a lower level of education and over half (57.5%) were unemployed, whereas over half of the fathers (61.3%) had a lower level of education and almost half (45.3%) were unemployed (see also table 1).

**Table 1:** Differences in socio-demographic characteristics between pregnancy and non-pregnancy groups.

**Socio-demographics **(total N)	**Total group**		**Pregnancy**		**Non-pregnancy**		**P**
	**N**	**%**	**N**	%	**N**	**%**	
civil law measure (206)*	167	81.1	35	85.4	132	80.0	0.433
father low education (212)	130	61.3	22	51.2	108	63.9	0.126
father unemployed (212)	96	45.3	22	51.2	74	43.8	0.386
mother low education (212)	103	48.6	25	58.1	78	46.2	0.160
mother unemployed (212)	122	57.5	25	58.1	79	57.4	0.930
history of closed placements (196)	69	35.2	14	35.9	55	35.0	0.919
history of residential placements (194)	140	72.2	29	78.4	111	70.7	0.349
history of foster care (196)	33	16.8	9	23.1	24	15.3	0.245
history of outpatient care(190)	141	74.2	31	79.5	110	72.8	0.398
Dutch ethnicity (208)	119	57.2	28	66.7	91	54.8	0.166

*total N varies due to missing files

### Procedure

The project was approved by the review boards of the Ministry of Justice, which imposed strict conditions in terms of confidentiality, appropriate handling of information and the participants' assent for participation and for contacting the parents. Shortly after admission (within one week), all eligible girls were approached individually by the interviewers in order to explain the purpose of the study. It was explained and written on the consent form that participation was voluntary, that refusal would not affect their legal status and that confidentiality was guaranteed. Participants were by no means forced to participate. The participants signed a consent form before the study commenced. The parents or primary caregivers were informed by letter. Parents could object to their daughter's participation, which only occurred for one participant. The consent procedure was carried out at least one week before the assessment. The instruments were presented and completed in a fixed order. First, participants were asked to fill in self-report questionnaires in groups of 3 girls at a time, and subsequently, the interview was carried out individually, preferably on the same day. When administering self-report questionnaires, a researcher was present and available for questions.

### Measures

#### File information

Information on socio-demographic background: the parents' occupation and educational background, age and ethnicity, and judicial measures, past detention and past residential placements, history of foster care and outpatient care was obtained from the institution file by means of a checklist. Information on contraception, medication, and method of pregnancy termination were gathered from the medical file.

Information on sexually-transmitted diseases (STDs) (lifetime) was gathered from the medical file as an indication of sexual risk behavior.

#### Social and Health Assessment (SAHA)

The Social and Health Assessment (SAHA) [26,27] was used to assess pregnancy, sexual risk behavior, multiple sex partners, early menarche and lack of assertiveness in sexual matters. The following SAHA items were used as measures of sexual risk behavior: use of contraception (condom use at last intercourse, use of contraceptives at last intercourse), and substance use at last intercourse. Sexual risk behavior was considered present if the participants answered positive to one of the following items: no condom use, no or insufficient use of other forms of contraception, substance use at last intercourse, or if a history of STDs was found in the file. In our sample we defined early menarche as having started before the age of 12. Lack of assertiveness in sexual matters was based on two questions in the SAHA: "how difficult would it be for you to use a condom every time you have sex?" and "how difficult would it be for you to tell your partner you don't want to have sex?" (response options: easy or difficult).

#### Kiddie-SADS present and lifetime version (Kiddie-SADS-P-L)

Conduct disorder, alcohol use disorder, drug use disorder and suicidality (based on one or more suicidal symptoms or attempts) were assessed by means of the K-SADS-P-L [28,29], a semi-structured interview on psychiatric disorders listed in the *Diagnostic and Statistical Manual of Mental Disorders-IV *[30]. The assessment was carried out by four experienced clinicians. Test-retest reliability for the various disorders assessed by means of the Kiddie-SADS has been described as good to excellent and concurrent validity and inter-rater agreement was reported to be high [31,32]. The introductory interview was left out because most items were administered by means of an introductory interview on socio-demographic characteristics and aspects of daily functioning, largely overlapping with the Kiddie-SADS content. The scores on the Kiddie-SADS were dichotomized in 0: diagnosis not present (answers 0: no information and 1: diagnosis not present) and 1: present in a moderate or severe form.

#### Sexual trauma

Information on sexual trauma (lifetime) was derived from a self-report questionnaire on trauma, translated and adapted from the "Traumatic Events Screening Inventory" (TESI-C; National Center for PTSD, 1996), in which one question assessed whether the participant had ever been involuntarily sexually approached or abused by someone more than five years older (answer options: yes or no). If the participant responded positive on this question, the age at the time of the sexual trauma and the frequency was asked for.

### Statistical analysis

The SPSS (Statistical Package for Social Sciences, version 11.0) statistical program has been used for analyzing the data. First, descriptive statistics were provided on pregnancy. Second, individuals from the pregnancy group and the non-pregnancy group were compared in terms of sexuality related factors and other risk factors (socio-demographic and mental health characteristics) using Chi-square tests (Fisher Exact when expected cell counts less than 5). The level of statistical significance (two sided) was set at .05. Third, all factors shown in tables 2 and 3 with a p-value < 0.1 (sexual risk behavior, sexual trauma, multiple sex partners and early maturity and drug use disorder, suicidality, and age) were incorporated as potential predictors in the multiple logistic regression analysis with pregnancy as the dependent variable. The forward method was used (adding variables one-by-one). The odds-ratios represented show how much more likely the presence of these factors is in the pregnancy group as compared to the non-pregnancy group, adjusted for the other variables in the model.

**Table 2:** Differences in sexuality related characteristics between pregnancy and non-pregnancy groups.

**Variables (total N)**	**Total group**		**Pregnancy**		**Non-pregnancy**			
	**N**	**%**	**N**	**%**	**N**	**%**	**Odds ratio**	**95% CI**
sexual risk behavior (212)	108	50.9	32	74.4	76	45.0	3.6	1.68–7.53**
sexual trauma (204)	103	50.5	28	66.7	75	46.3	2.3	1.14–4.73**
multiple sex partners (209)	76	36.4	24	55.8	52	31.3	2.8	1.39–5.50**
lack of assertiveness (202)	24	11.9	3.0	7.1	21	13.1	2.0	0.56–6.93
early maturity (193)	75	38.9	21	51.2	54	35.5	1.9	0.95–3.8*

**significant at the 0.05 level

*also included in the regression because of p < 0.1

**Table 3:** Differences in mental health characteristics between pregnancy and non-pregnancy groups.

**Variables (total N)**	**Total group**		**Pregnancy**		**Non-pregnancy**			
	**N**	**%**	**N**	**%**	**N**	**%**	**Odds ratio**	**95% CI**
conduct disorder (203)	111	54.7	27	64.3	84	52.2	1.7	0.82–3.33
alcohol use disorder(203)	40	19.7	11	25.6	29	18.1	1.6	0.70–3.44
drug use disorder(203)	107	52.7	28	65.1	79	49.4	1.9	0.95–3.85*
suicidality (204)	129	63.2	32	74.4	97	60.2	1.9	0.90–4.08*

*also included in the regression because of p < 0.1

## Results

### Lifetime rates of pregnancy and comparison of the pregnancy and the non-pregnancy groups

We divided our sample into two groups: a pregnancy group (N = 43, 20%) and a non-pregnancy group (N = 169, 80%). Twenty percent of the participants reported one or more pregnancies ever, while none of the girls had actually given birth to a child. No information was found on specific method of termination of pregnancy in the files. Medical files also hardly revealed miscarriages or abortions, abortions were mentioned only in 7 cases.

The ages of the total group ranged from 12 to 19, (mean age 15.57; SD = 1.39). The mean age of the girls in the pregnancy group (16.07; SD 1.39) was significantly higher than the girls in the non-pregnancy group (15.45; SD 1.31; p = 0.009). In table 1 other sociodemographic characteristics of the pregnancy and the non-pregnancy group are shown, such as judicial measure, level of education and employment of the parents, history of placements, history of foster care and outpatient care, and ethnicity. None of these characteristics differed significantly between the pregnancy and the non-pregnancy groups. Total IQ didn't differ either between both groups (pregnancy group:mean IQ: 88.5 SD 15.4;non-pregnancy group: mean IQ: 88.7 SD 15.6; p = 0.934).

Differences in sexuality related and mental health related characteristics between pregnancy and non-pregnancy groups are shown in tables 2 and 3. A number of sexuality related characteristics differed between the pregnancy group and the non-pregnancy group; sexual risk behavior, multiple sex partners and sexual trauma were more prevalent in the pregnancy group than in the non-pregnancy group. There was no difference between groups in (lack of) assertiveness in sexual issues. In the medical files only in 17 cases use of oral contraceptives was mentioned. By self-reports (N = 206) 25 girls (12.1%) mentioned no or insufficient use of contraception the last time they had sex, 7 (17.1%) were in the pregnancy group and 18 (10.9%) in the non-pregnancy group (p = 0.279). Early maturity showed a trend (p < 0.1) towards being significantly higher in the pregnancy group. As for the mental health characteristics, drug use disorder and suicidality showed a trend in the same direction. There were no significant differences between the groups in terms of conduct disorder and alcohol use disorder. In the medical files only in 13 cases use of methylfenidate was mentioned.

### Predictors of pregnancy

In table 4 the predictive value of risk factors for pregnancy are shown. Variables with p < 0.1 in tables 2 and 3 were included in the regression (i.e. suicidality, sexual risk, early maturity, age, drug use disorder, sexual trauma and multiple sex partners. It is shown that four variables, i.e. age, early maturity, sexual risk behavior and suicidality, predicted pregnancy group membership.

**Table 4:** Predictive value of various risk factors.

					**95% CI**	
	**B**	**SE**	**P**	**Odds-ratio**	**lower**	**upper**
suicidality	0.971	0.467	0.037	2.641	1.058	6.595
sexual risk	0.822	0.443	0.064	2.275	0.954	5.424
early maturity	0.887	0.415	0.032	2.428	1.077	5.476
age	0.453	0.156	0.004	1.573	1.159	2.135

also included in the regression analysis: drug use disorder, sexual trauma, and multiple sex partners

## Discussion

This study confirms high prevalence rates of teenage pregnancy in adolescent female detainees. The prevalence of about 20% is high like the percentages found in North American detainees. Neglected, traumatized and abused girls may be more at risk of being detained, while such history also predisposes to sexual risk behavior. None of the girls had actually given birth to a child. Although abortions were only mentioned in 7 medical files, it is very likely that most of the pregnancies ended in abortions, as in the Netherlands abortion is a legal and accessible way of pregnancy termination.

The differences between the pregnancy and non-pregnancy groups in terms of current age, sexual risk behavior and sexual trauma are consistent with previous research among North American girls [13-15,33-37]. Suicidality and early maturity as factors associated with teenage pregnancy (both showing a trend towards significance) also confirm earlier research among adolescent females [22-25]. However, unlike other studies [4-11] this study did not show differences between groups regarding alcohol use disorder or conduct disorder.

Of all factors used in the regression, higher age, sexual risk behavior, early maturity and suicidality were the best predictors of pregnancy. It is not surprising that sexual risk behavior and age are predictors of pregnancy. Sexual activity increases with age, and some aspects of risky sexual interaction (e.g. not using contraception at intercourse) are a primary cause of pregnancy. Our finding on early maturity has also been reported earlier. Again, one would expect early maturers to be sexually active at a younger age, which may subsequently increase the risk of early and unwanted pregnancies. However, the relationship between teenage pregnancy and suicidality has not been reported earlier.

Suicidality, sexual risk behavior and drug use might well be part of impulsivity in a developing Cluster B personality disorder. A current follow-up study has included a personality screening.

In summary, our findings indicate that high numbers of detained adolescent females become pregnant in (early) adolescence. In this respect the Dutch situation is not much different from the situation among North American detainees, despite the extensive sex education given at Dutch schools. This unfortunate situation may be linked to many factors, making it necessary to incorporate a wide range of factors in prevention and intervention programs for this population, e.g. programs focused on prevention of sexual risk behavior, but also on suicidality intervention.

## Conclusion

### Clinical implications

The lifetime prevalence of teenage pregnancy among detained girls is high and associated with both sexuality related characteristics and mental health characteristics. Therefore, the diagnostic assessment of detained adolescent females should be comprehensive and include adequate psychological and psychiatric assessment as well as a comprehensive assessment of sexual risk. Clinicians should realize that a history of teenage pregnancy could indicate a certain combination of risk factors. Future research should evaluate whether intervention programs will result in a reduction of teenage pregnancy in this sample.

### Limitations

Some limitations of this study should be mentioned. First, only self-report information was available for most participants. Sexuality is a sensitive topic and it is conceivable that subjects, consciously or unconsciously, have provided social desirable answers (e.g. regarding assertiveness in sexual matters). Secondly, the cross-sectional nature of the study did not allow us to investigate causal pathways between possible risk factors and pregnancy. For this purpose, longitudinal studies assessing adolescent females before and after detention should be conducted. Thirdly, we were not able to compare groups on education or time in residential care. We forwent comparisons on psychopathological comorbidity as this was described in another publication focusing on psychopathology and aggression (Hamerlynck et al, 2007, in press). A relevant finding in this respect was that 20.8% of the girls had a diagnosis of ADHD.

Finally, it is unknown whether these findings can be generalized to detained girls in other countries, as cross-cultural differences may exist. However, as mentioned above, many results approximate results reported in North American samples of detainees, so it is likely that, in these girls, risk factors for pregnancy are similar across Western countries.

## Competing interests

The author(s) declare that they have no competing interests.

## Authors' contributions

All authors participated in the design of the study and read and approved the final manuscript. SH and PB performed the statistical analysis.
